# Serum microRNA-1 and microRNA-133a levels reflect myocardial steatosis in uncomplicated type 2 diabetes

**DOI:** 10.1038/s41598-017-00070-6

**Published:** 2017-03-03

**Authors:** D. de Gonzalo-Calvo, R. W. van der Meer, L. J. Rijzewijk, J. W. A. Smit, E. Revuelta-Lopez, L. Nasarre, J. C. Escola-Gil, H. J. Lamb, V. Llorente-Cortes

**Affiliations:** 1 Biomedical Research Institute Sant Pau (IIB Sant Pau), Barcelona, Spain; 2 CIBERCV, Institute of Health Carlos III, Madrid, Spain; 3 Department of Radiology, Leiden University Medical Center, Leiden, The Netherlands; 4 Department of Medicine, Kantonsspital Baden AG, Baden, Switzerland; 5 Department of Internal Medicine, University Medical Center Nijmegen, Nijmegen, The Netherlands; 6 IIB Sant Pau, Departament de Bioquímica i Biologia Molecular, Universitat Autònoma de Barcelona-CIBER de Diabetes y Enfermedades Metabolicas Asociadas, Barcelona, Spain; 7 Biomedical Research Institute of Barcelona, CSIC, Barcelona, Spain

## Abstract

Using *in vitro*, *in vivo* and patient-based approaches, we investigated the potential of circulating microRNAs (miRNAs) as surrogate biomarkers of myocardial steatosis, a hallmark of diabetic cardiomyopathy. We analysed the cardiomyocyte-enriched miRNA signature in serum from patients with well-controlled type 2 diabetes and with verified absence of structural heart disease or inducible ischemia, and control volunteers of the same age range and BMI (N = 86), in serum from a high-fat diet-fed murine model, and in exosomes from lipid-loaded HL-1 cardiomyocytes. Circulating miR-1 and miR-133a levels were robustly associated with myocardial steatosis in type 2 diabetes patients, independently of confounding factors in both linear and logistic regression analyses (*P* < 0.050 for all models). Similar to myocardial steatosis, miR-133a levels were increased in type 2 diabetes patients as compared with healthy subjects (*P* < 0.050). Circulating miR-1 and miR-133a levels were significantly elevated in high-fat diet-fed mice (*P* < 0.050), which showed higher myocardial steatosis, as compared with control animals. miR-1 and miR-133a levels were higher in exosomes released from lipid-loaded HL-1 cardiomyocytes (*P* < 0.050). Circulating miR-1 and miR-133a are independent predictors of myocardial steatosis. Our results highlight the value of circulating miRNAs as diagnostic tools for subclinical diabetic cardiomyopathy.

## Introduction

In asymptomatic patients with type 2 diabetes, cardiac function, structure, and dimension are often altered, even in the absence of coronary artery disease or hypertension, due to diabetic cardiomyopathy^1^. The pathophysiology of non-ischemic diabetic cardiomyopathy is complex, and the precise underlying mechanisms are not fully understood. Several groups, including ours, have proposed abnormal regulation of lipid uptake or its intracellular metabolism in cardiomyocytes as one of the mechanisms underlying diabetic cardiomyopathy^2–4^. Myocardial steatosis, defined as the accumulation of neutral lipids in the myocardium, has been previously observed in diabetic patients with end-stage non-ischemic cardiomyopathy^5^. Myocardial steatosis is independently associated with impaired contractile function in both type 2 diabetes patients and animal models^6–10^. A causal relationship has been established between myocardial neutral lipid content and contractile function^11, 12^. Importantly, myocardial steatosis is an early manifestation in the pathogenesis of cardiac-related complications; this process precedes the onset of both diastolic and systolic dysfunction and is evident in the absence of heart failure^8, 10^. Thus, myocardial steatosis is clinically useful for the early identification and stratification of high-risk type 2 diabetes patients. However, clinical application of the gold standard for the evaluation of myocardial neutral lipid content, proton magnetic resonance spectroscopy (^1^H-MRS), is currently impractical for large-scale population screening. This technique is complex, expensive, causes patient discomfort, and requires specialized centres and personnel. The identification of blood biomarkers of myocardial steatosis could permit routine population-wide screening, allowing early diagnosis and anticipation of cardiac dysfunction.

Clinical studies have proposed peripheral blood microRNAs (miRNAs) as sensitive, specific and non-invasive biomarkers for the early monitoring of alterations in cardiac viability, structure, and function^13, 14^. Distinctive circulating miRNA signatures with clinical value have been described in coronary heart disease^15^, acute coronary syndrome^16^, heart failure^17^, various cardiomyopathies^18, 19^, and type 2 diabetes and its related vascular complications^20–25^. Nevertheless, the value of circulating miRNAs as biomarkers of cardiac dysfunction in type 2 diabetes remains unknown. We hypothesized that circulating miRNAs could be used to monitor myocardial neutral lipid content in type 2 diabetes. We explored the potential of cardiomyocyte-enriched miRNAs as surrogate biomarkers of myocardial steatosis in patients with well-controlled type 2 diabetes of short duration and with verified absence of cardiac structural heart disease or inducible ischemia, and in a high-fat diet-fed murine model of insulin resistance. We also analysed the cardiomyocyte-enriched miRNA signature in exosomes released from neutral lipid-loaded HL-1 cardiomyocytes. The results obtained from patients and both *in vivo* and *in vitro* models suggest that serum miR-1 and miR-133a levels hold significant promise as clinical indicators of myocardial steatosis.

## Results

### Characteristics of study population

Table 1 shows the characteristics of the type 2 diabetes population. Data from the PIRAMID study have already been published elsewhere^26, 27^. The typical study participant was a Caucasian male with a mean ± SD age of 56.5 ± 5.6 years, diabetic (fasting glucose level = 8.6 ± 1.9 mmol/L and HbA_1c_ = 7.1 ± 1.0%), slightly overweight (BMI = 28.7 ± 3.4 kg/m^2^) and prehypertensive (SBP = 130.0 ± 12.1 mmHg and DBP = 81.6 ± 8.1 mmHg). The absence of ischemia and structural abnormalities was verified in all patients. Myocardial steatosis data, ranging from 0.14% to 2.18%, was available for 74 patients (95%).

**Table 1:** Characteristics of the uncomplicated type 2 diabetes patients.

Variable	N = 72
***Clinical Characteristics***	
Age (years)	56.5 ± 5.6
Male N (%)	72 (100)
Time since diagnosis of diabetes (years)	4.1 ± 2.7
Current smoker N (%)	15 (20.8)
Body mass index (kg/m^2^)	28.7 ± 3.4
Waist circumference (cm)	104.4 ± 10.2
Concomitant medication N (%)	
Statin	35 (48.6)
Any antihypertensive medication	32 (44.4)
β-Blocker	7 (9.7)
Diuretic	11 (15.3)
ACE inhibitor	18 (25.0)
ARB	8 (11.1)
Calcium antagonist	4 (5.6)
***Biochemical and Metabolic Characteristics***	
HbA_1c_ (%)	7.1 ± 1.0
Plasma fasting glucose (mmol/L)	8.6 ± 1.9
Plasma fasting insulin (pmol/L)	72.5 ± 37.1
Visceral fat volume (mL)	434.5 ± 203.2
Subcutaneous fat volume (mL)	681.7 ± 255.1
Total cholesterol (mmol/L)	4.7 ± 0.9
LDL-cholesterol (mmol/L)	2.8 ± 0.8
HDL-cholesterol (mmol/L)	1.1 ± 0.3
Non-HDL-cholesterol (mmol/L)	3.6 ± 1.0
Plasma triglycerides (mmol/L)	1.8 ± 1.1
Plasma NEFA (mmol/L)	0.5 ± 0.2
Leukocyte count (×10^3^/μL)	6.2 ± 1.8
us-CRP (mg/L)	8.6 ± 19.5
NT-proBNP (ng/L)	35.9 ± 27.6
***Hemodynamic Parameters and Cardiac Dimensions and Function***	
Systolic blood pressure (mm Hg)	130.0 ± 12.1
Diastolic blood pressure (mmHg)	81.6 ± 8.1
Heart rate (bpm)	65.6 ± 8.7
LV mass (g)	107.6 ± 17.0
LV end-systolic volume (mL)	63.0 ± 15.0
LV end-diastolic volume (mL)	157.0 ± 25.2
Stroke volume (mL)	93.9 ± 16.3
Ejection Fraction (%)	60.0 ± 5.7
Cardiac index (L/min*m^2^)	2.0 ± 0.8
E peak filling rate (mL/s)	415.6 ± 84.3
E-dec_peak_ (mL/s^2^ × 10^−3^)	−3.5 ± 1.0
E-dec_mean_ (mL/s^2^ × 10^−3^)	−2.3 ± 0.7
E/A peak flow	1.0 ± 0.2
E/Ea	9.9 ± 3.9
Myocardial steatosis (%)	0.8 ± 0.4

Data are presented as mean ± SD for continuous variables and as frequencies (percentages) for categorical variables.

ACE: Angiotensin-converting enzyme; ARB: angiotensin receptor blocker; HbA_1c_: Glycated haemoglobin. For other abbreviations see the text.

### Confounding potential of the clinical characteristics of uncomplicated type 2 diabetes patients on circulating cardiomyocyte-enriched miRNAs

Circulating miRNA levels were available for 72 of the 74 samples analysed (97%). After the RNA isolation procedure, two samples were found to be of poor quality and were excluded from further analyses. miR-1, miR-133a, and miR-133b levels were below the limit of detection in 22.2%, 13.9%, and 4.2% of patients, respectively. These variables were entered into univariate and multivariate linear regression models after log transformation to account for skewed data distribution. miR-208a, miR-208b, and miR-499 were below the limit of detection in 84.7%, 84.7% and 80.6% of patients, respectively, and were not considered for further analysis.

To determine the extent to which serum miRNA levels are influenced by the characteristics of the study population, we evaluated the association between clinical parameters and levels of miR-1, miR-133a, and miR-133b (Table 2). Univariate regression analysis revealed an inverse association between miR-1 levels and the E/Ea ratio (β = −0.305, *P* = 0.030). A borderline association between miR-1 and LV mass was observed (β = −0.243, *P* = 0.071). Circulating miR-133a levels were directly associated with plasma triglyceride concentration (β = 0.263, *P* = 0.041). A near-significant association was also observed between miR-133a and HDL-C (β = −0.213, *P* = 0.099). No association was observed between miR-133b and other clinical parameters. There was no statistically significant association between serum levels of the miRNAs measured and medications, except in the case of miR-133b, the levels of which tended to be higher in patients on antihypertensive medication (*P* = 0.061).

**Table 2:** Associations between serum cardiac-enriched miRNAs and characteristics of study population.

	miR-1		miR-133a		miR-133b	
	β	*P* -value	β	*P* -value	β	*P*-value
***Clinical Characteristics***						
Age (years)	−0.106	0.438	−0.105	0.415	−0.076	0.533
Time since diagnosis of diabetes (years)	0.077	0.574	−0.114	0.378	0.108	0.378
Body mass index (kg/m^2^)	−0.076	0.575	0.105	0.416	0.027	0.827
Waist circumference (cm)	−0.146	0.283	0.053	0.680	−0.036	0.767
***Biochemical and Metabolic Characteristics***						
HbA_1c_ (%)	−0.013	0.922	0.065	0.614	−0.023	0.853
Plasma fasting glucose (mmol/L)	−0.147	0.280	0.048	0.709	0.094	0.444
Plasma fasting insulin (pmol/L)	0.057	0.678	0.145	0.263	−0.027	0.826
Visceral fat volume (mL)	0.022	0.872	0.149	0.247	−0.003	0.979
Subcutaneous fat volume (mL)	−0.094	0.491	−0.028	0.830	0.066	0.591
Total cholesterol (mmol/L)	0.012	0.931	−0.010	0.942	−0.163	0.185
LDL-cholesterol (mmol/L)	−0.021	0.881	−0,115	0.386	−0.135	0.278
HDL-cholesterol (mmol/L)	−0.138	0.316	−0.213	0.099	0.021	0.865
Non-HDL-cholesterol (mmol/L)	0.045	0.746	0.043	0.744	−0.161	0.190
Plasma triglycerides (mmol/L)	0.094	0.495	0.263	0.041*	−0.114	0.354
Plasma NEFA (mmol/L)	0.033	0.813	0.024	0.855	0.186	0.132
Leukocyte count (x 10^3^/μL)	0.036	0.797	0.064	0.625	0.011	0.932
us-CRP (mg/L)	−0.038	0.784	−0.021	0.874	0.018	0.887
NT-proBNP (ng/L)	−0.146	0.282	−0.080	0.537	−0.122	0.316
***Hemodynamic Parameters and Cardiac Dimensions and Function***						
Systolic blood pressure (mm Hg)	−0.117	0.392	0.000	0.999	0.019	0.874
Diastolic blood pressure (mmHg)	0.053	0.697	0.078	0.548	0.051	0.679
Heart rate (bpm)	0.029	0.829	−0.170	0.187	0.055	0.655
LV mass (g)	−0.243	0.071	−0.057	0.663	−0.047	0.700
LV end-systolic volume (mL)	−0.069	0.616	−0.052	0.688	0.197	0.105
LV end-diastolic volume (mL)	−0.018	0.896	0.038	0.771	0.151	0.215
Stroke volume (mL)	0.039	0.774	0.106	0.412	0.054	0.660
Ejection fraction (%)	0.125	0.359	0.117	0.366	−0.118	0.334
Cardiac index (L/min*m^2^)	0.169	0.214	−0.001	0.993	0.065	0.596
E peak filling rate (mL/s)	0.090	0.511	−0.027	0.836	0.100	0.412
E-dec_peak_ (mL/s^2^ × 10^−3^)	−0.079	0.564	−0.102	0.431	0.055	0.656
E-dec_mean_ (mL/s^2^ × 10^−3^)	−0.079	0.565	−0.033	0.796	0.053	0.666
E/A peak flow	0.095	0.484	0.090	0.485	−0.016	0.899
E/Ea	−0.305	0.030*	0.017	0.898	0.084	0.510

Data are expressed as standardized beta (β).

### Circulating cardiomyocyte-enriched miR-1 and miR-133a levels are independent predictors of myocardial steatosis in patients with type 2 diabetes

Linear regression analysis was performed to study the associations between myocardial steatosis and circulating cardiomyocyte-enriched miRNAs (Table 3). Univariate analysis (model 1) revealed a direct association between circulating levels of miR-1 (β = 0.360, *P* = 0.006) and miR-133a (β = 0.335, *P* = 0.008), but not miR-133b (β = 0.157, *P* = 0.198). Multivariate analysis was performed to explore in detail the relationship between myocardial neutral lipid content and miR-1 and miR-133a levels (Table 3). In model 2, myocardial steatosis was entered as a dependent variable and age, visceral fat volume, non-HDL-cholesterol, plasma TG, and miR-1 or miR-133a levels were subsequently entered as independent variables. Possible confounders included plasma fasting glucose, plasma fasting insulin, BMI, HDL-cholesterol, plasma NEFA, NT-proBNP (N-terminal pro b-type natriuretic peptide), us-CRP, LV mass, ejection fraction, and E/E_a,_ were separately entered as independent variables into model 2 (model 3). Adjustment for different clinical, biochemical, metabolic or cardiac parameters had no effect on the association between myocardial steatosis content and circulating miR-1 or miR-133a levels (*P* < 0.010 for all models). The observed associations also remained statistically significant after correction for multiple comparisons. In healthy volunteers, no association between myocardial steatosis, ranging from 0.34% to 1.05%, and serum miRNA levels was observed (*P* > 0.050 for all associations).

**Table 3:** Association between myocardial steatosis and serum cardiomyocyte-enriched miRNA in patients with uncomplicated type 2 diabetes.

	β	*P*-value		β	*P*-value
**Model 1**			**Model 1**		
miR-1	0.360	0.006	miR-133a	0.335	0.008
**Model 2**			**Model 2**		
miR-1	0.371	0.004	miR-133a	0.349	0.006
Age	0.229	0.069	Age	0.277	0.024
Visceral fat volume	0.139	0.277	Visceral fat volume	0.106	0.389
Non-HDL-cholesterol	0.233	0.156	Non-HDL-cholesterol	0.272	0.080
Plasma triglyceride	0.008	0.963	Plasma triglyceride	−0.045	0.775
**Model 3**			**Model 3**		
Association between myocardial steatosis and serum miR-1 for model 2 and each of following variables:			Association between myocardial steatosis and serum miR-133a for model 2 and each of following variables:		
i) Plasma fasting glucose	0.387	0.003	i) Plasma fasting glucose	0.352	0.006
ii) Plasma fasting insulin	0.370	0.004	ii) Plasma fasting insulin	0.355	0.006
iii) BMI	0.384	0.003	iii) BMI	0.349	0.007
iv) HDL-cholesterol	0.366	0.005	iv) HDL-cholesterol	0.346	0.007
v) Plasma NEFA	0.411	0.001	v) Plasma NEFA	0.355	0.007
vi) NT-proBNP	0.378	0.004	vi) NT-proBNP	0.349	0.007
vii) us-CRP	0.425	0.001	vii) us-CRP	0.401	0.002
viii) LV mass	0.351	0.009	viii) LV mass	0.337	0.009
ix) Ejection fraction	0.339	0.008	ix) Ejection fraction	0.333	0.009
x) E/Ea	0.413	0.005	x) E/Ea	0.385	0.006

Data are expressed as standardized beta (β).

Myocardial steatosis has been reported as significantly higher in type 2 diabetes patients compared to healthy subjects^10^. To further confirm the close relationship between myocardial steatosis and circulating miR-1 and miR-133a levels, serum levels of both miRNAs were measured in 12 patients with uncomplicated type 2 diabetes and in 12 healthy volunteers matched for age and BMI (Supplementary Information, Table S1). Serum miR-133a levels were increased in uncomplicated type 2 diabetes patients as compared with healthy subjects (type 2 diabetes group = 1.28 ± 0.34, control group = 0.93 ± 0.15; *P* = 0.013). No differences in serum miR-1 levels were observed between groups (type 2 diabetes group = 1.33 ± 0.35, control group = 1.01 ± 0.36; *P* = 0.073).

**Figure 1:**
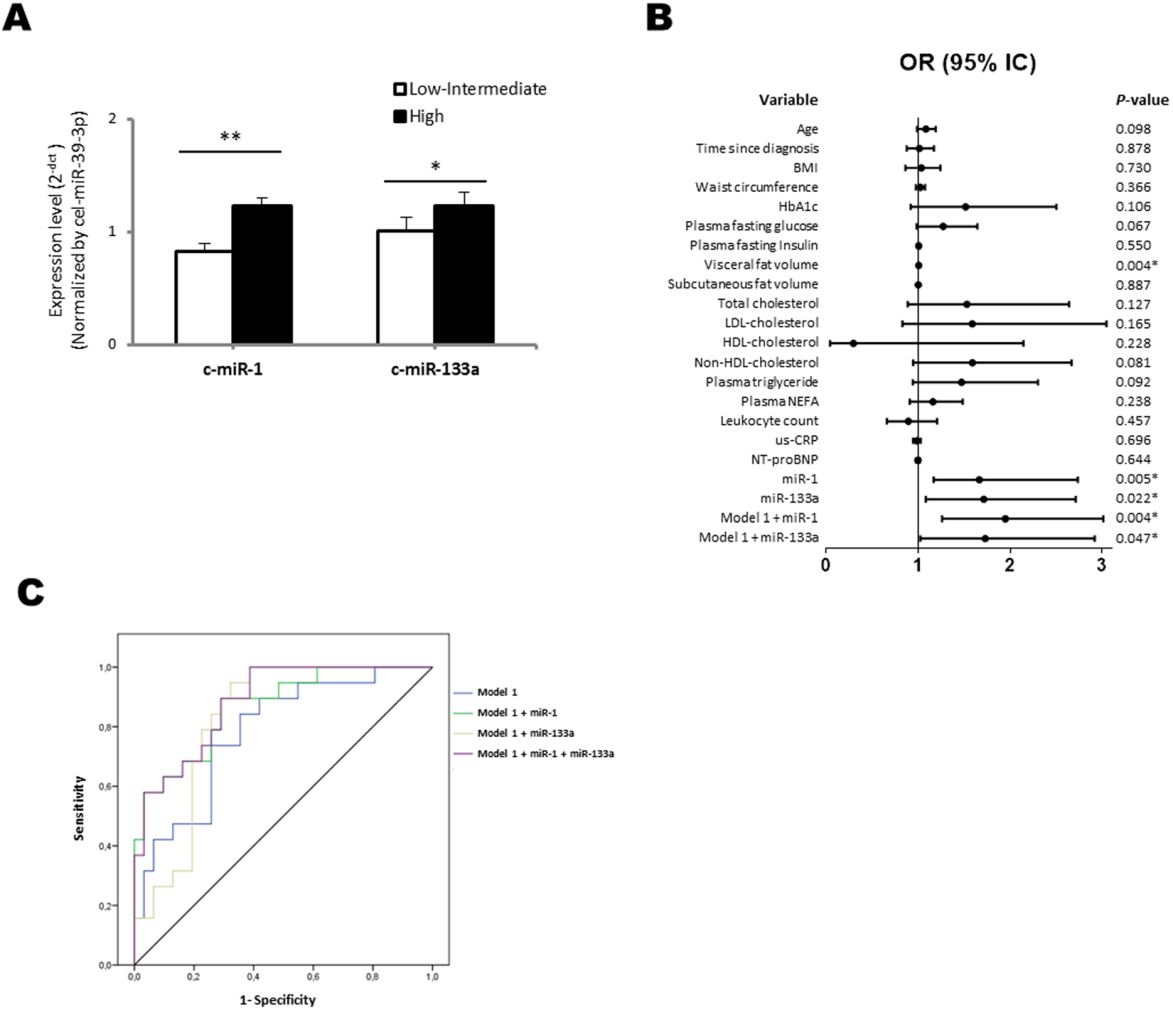
(**A**) Quantification by RT-qPCR of serum miR-1 and miR-133a levels in patients with uncomplicated type 2 diabetes in tertiles 1 and 2 (low-intermediate levels; N = 48) or 3 (high levels; N = 24) of myocardial steatosis. Relative quantification was performed using cel-miR-39-3p for normalization. Differences between groups were analysed using a Student’s t-test for independent samples. Data represent the mean + SD. *P < 0.050; **P < 0.010. (**B**) Association between myocardial steatosis and clinical parameters or serum cardiomyocyte-enriched miRNAs in patients with uncomplicated type 2 diabetes. Univariate and multivariate logistic regression models were used to explore the association between serum cardiomyocyte-enriched miRNA and myocardial steatosis as outcome. OR: Odds Ratio, CI: Confidence Interval. (**C**) ROC curves for the logistic regression models of myocardial steatosis. Model 1: age, plasma fasting glucose, visceral adipose tissue, plasma TG and non-HDL-cholesterol.

Additional analyses were performed to evaluate the potential of circulating miR-1 and miR-133a as biomarkers of myocardial steatosis. Since there is not an established clinical range for myocardial steatosis, we stratified our study population in two study groups: those type 2 diabetes patients with high levels of myocardial steatosis (patients in tertile 3 of myocardial steatosis, ranging from 0.93% to 2.18%; N = 24) and those type 2 diabetes patients with low-intermediate levels of myocardial steatosis (patients in tertiles 1 and 2 of myocardial steatosis, ranging from 0.14% to 0.92%; N = 48). Type 2 diabetes patients in the third tertile of myocardial steatosis (high levels) showed a higher level of circulating miR-1 and miR-133a than those tertiles 1 and 2 (low-intermediate levels) (Fig. 1A). Using logistic regression models, we explored the association between the levels of myocardial steatosis and different potential predictors, including miR-1 and miR-133a. As shown, in Fig. 1B, we observed a direct association between myocardial steatosis and circulating miR-1 and miR-133a. An association with the content of visceral adipose tissue was also observed. No association was reported for other clinical parameters. Interestingly, adjustment for different clinical variables, including age, plasma fasting glucose, visceral adipose tissue, plasma TG and non-HDL-cholesterol (model 1), had no effect on the association observed between myocardial steatosis and both miRNAs (Fig. 1B).

Owing the close association between myocardial steatosis and the serum levels of miR-1 and miR-133a, and to further explore the possible role of both miRNAs as biomarkers, we evaluated whether miR-1 and miR-133a improved the discrimination of a model of myocardial steatosis. To do that, we analysed four logistic regression models: (i) model 1: clinical variables statistically associated, or close to be statistically associated, with myocardial steatosis, including age, plasma fasting glucose, visceral fat volume, non-HDL-cholesterol and plasma triglyceride; (ii) model 2: model 1 + miR-1; (iii) model 3: model 1 + miR-133a; (iv) model4: model 1 + miR-1 + miR-133a. Figure 1C shows the receiver operating characteristic (ROC) analysis of all models. The highest area under the ROC curve (AUC) was observed for the model containing the clinical parameters and both miR-1 and miR-133a [AUC (95% CI) = 0.783 (0.654–0.912) for model 1; 0.866 (0.765, 0.967) for model 2; 0.825 (0.710–0.940) for model 3; 0.883 (0.793–0.972) for model 4]. Sensitivity and specificity were 73.7% and 74.2%, for model 1, 78.9% and 71.0% for model 2, 78.9% and 74.2% for model 3 and 78.9% and 77.4% for model 4.

### Levels of circulating cardiomyocyte-enriched miR-1 and miR-133a are increased in an *in viv*o mouse model of high-fat diet-induced insulin resistance

**Figure 2:**
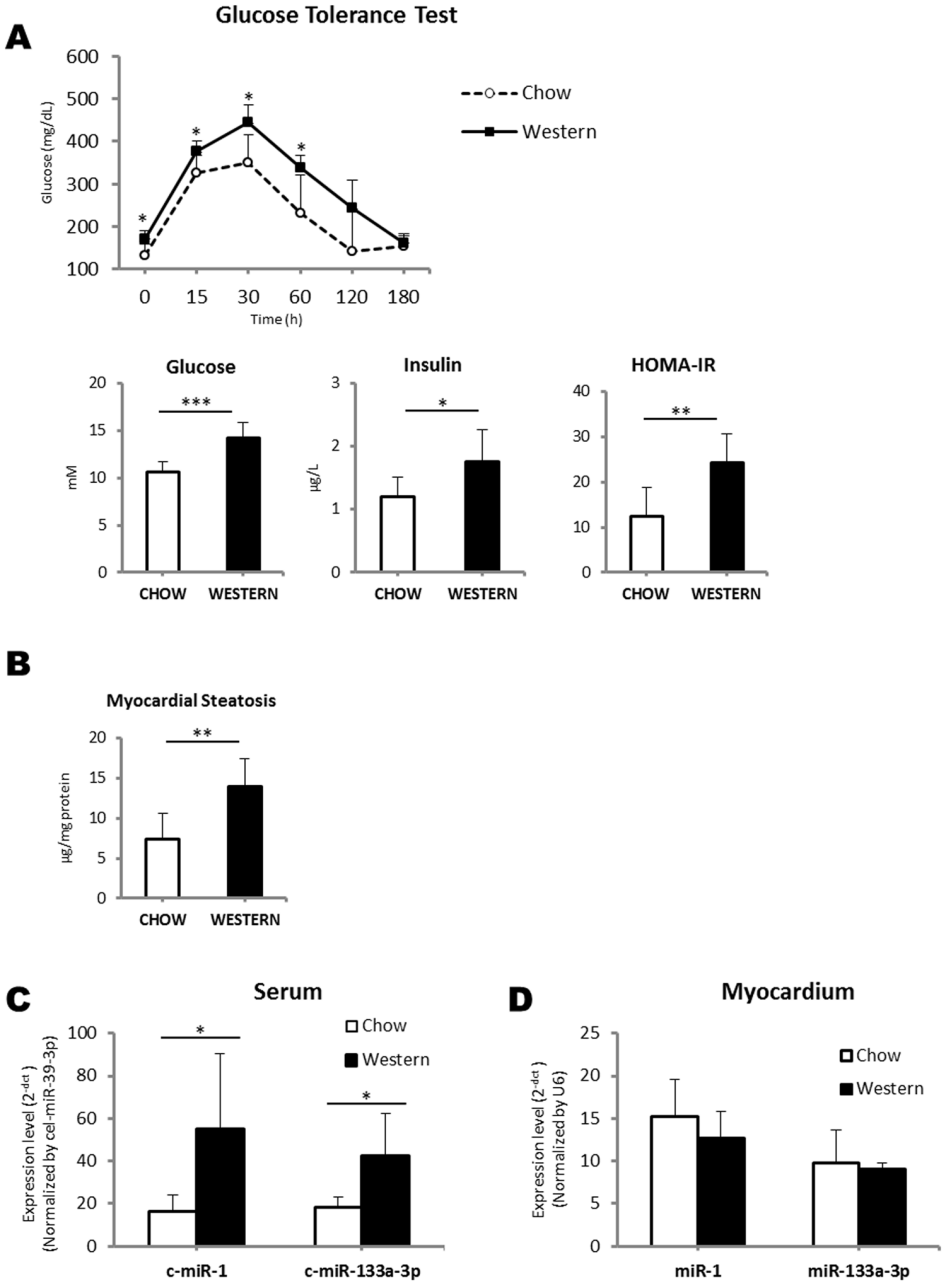
(**A**) Metabolic parameters in mice fed with chow (N = 6) vs those fed on a Western diet (N = 6): glucose metabolism after 6 hours of fasting followed by intraperitoneal injection of glucose (2 g/kg BW); fasting plasma glucose levels; fasting plasma insulin levels; insulin-resistance index (HOMA-IR). (**B**) Myocardial steatosis in mice fed with chow vs those fed on a Western diet. (**C**) Quantification by RT-qPCR of serum miR-1 and miR-133a levels in mice fed with chow vs those fed on a Western diet. Relative quantification was performed using cel-miR-39-3p for normalization. (**D**) Quantification by RT-qPCR of myocardial miR-1 and miR-133a levels in mice fed with chow vs those fed on a Western diet. Relative quantification was performed using U6 for normalization. Differences between groups were analysed using a Student’s t-test for independent samples. Data represent the mean + SD. *P < 0.050; **P < 0.010; ***P < 0.001.

To validate the association between myocardial neutral lipid accumulation and circulating cardiomyocyte-enriched miR-1 and miR-133a levels, we tested our clinical findings in an animal model of insulin resistance induced by a high-fat diet. Body composition and biochemical parameters for both groups of animals are shown in Table S2 (Supplementary Information). Six weeks of Western diet significantly increased fasting plasma glucose and insulin levels, as well as the insulin resistance index (HOMA-IR) (Fig. 2A). A glucose tolerance test in mice fed on a Western diet revealed delayed glucose clearance as compared with those fed on a chow diet. As expected, the accumulation of neutral lipid content in the myocardium was significantly higher in mice fed on a Western diet as compared with those on a normal chow diet (Fig. 2B). No differences in cardiac function parameters were observed between study groups (p < 0.050 for all parameters).

After demonstrating an increase in myocardial neutral lipid accumulation in our murine model of insulin resistance, we next analysed levels of miR-1 and miR-133a in the serum and myocardium of both diet groups. Corroborating our clinical findings, we observed increases in serum levels of miR-1 (3.3-fold) and miR-133a (2.4-fold) in the Western diet group with respect to the chow diet group (Fig. 2C). Myocardial levels of miR-1 and miR-133a were similar in both groups (Fig. 2D). A correlation between the myocardial neutral lipid content and circulating levels of miR-1 and miR-133a was observed (ρ = 0.622, *P* = 0.031, ρ = 0.755, *P* = 0.005, respectively).

### Circulating cardiomyocyte-enriched miR-1 and miR-133a are released by HL-1 cardiomyocytes following intracellular neutral lipid accumulation

**Figure 3:**
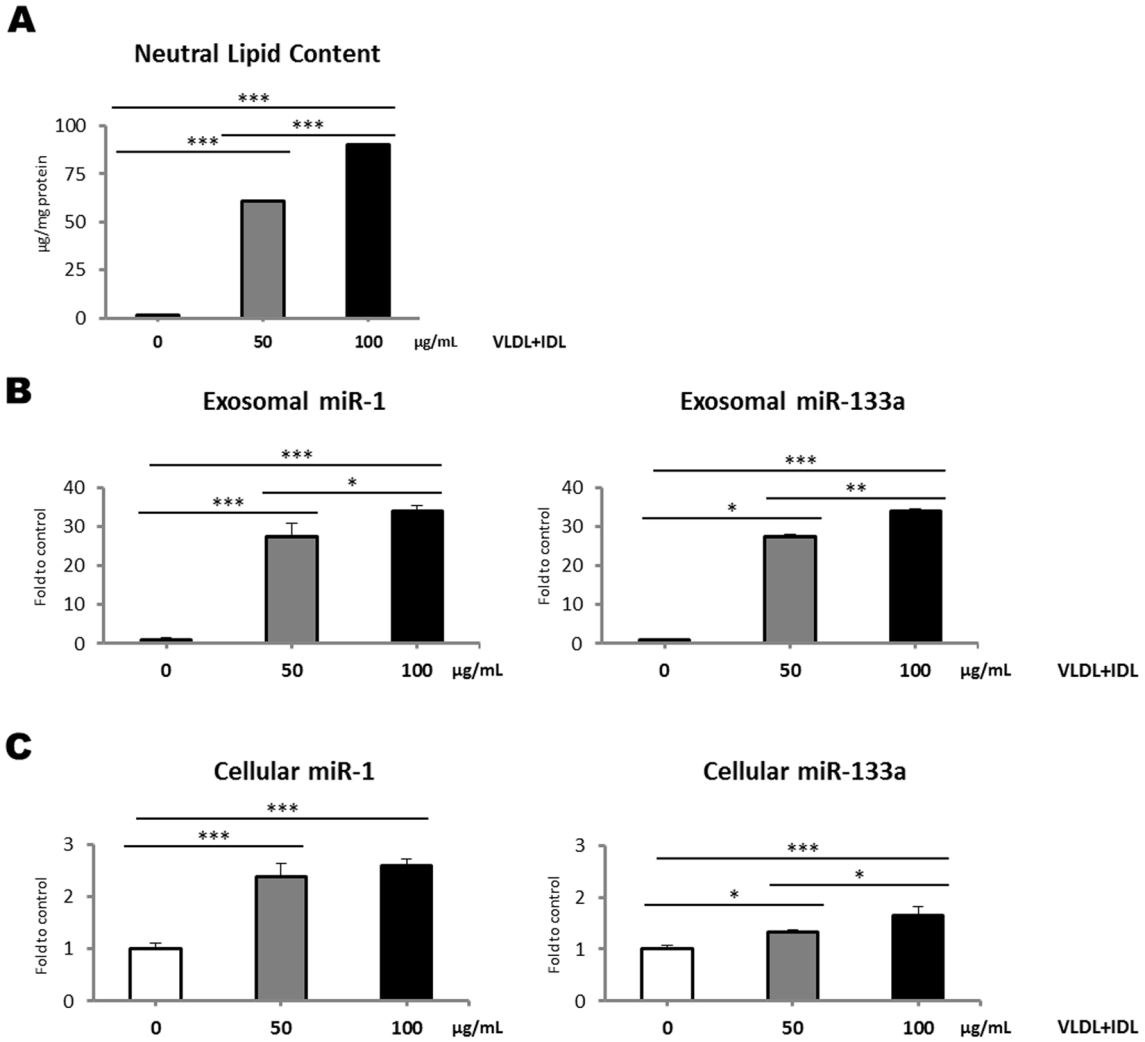
(**A**) Neutral lipid accumulation in HL-1 cells after exposure to VLDL+IDL. Data represent the mean ± SD. (**B**) Quantification by RT-qPCR of exosomal miR-1 and miR-133a levels after exposure to VLDL+IDL. Relative quantification was performed using cel-miR-39-3p for normalization. (**C**) Quantification by RT-qPCR of HL-1 miR-1 and miR-133a levels after exposure to VLDL+IDL. Relative quantification was performed using U6 for normalization. HL-1 cells were incubated for 24 hours in the absence or presence of VLDL+IDL (50 or 100 μg/mL). Differences between groups were analysed using one-way ANOVA followed by Tukey’s *post hoc* test for comparison between each subgroup. Results are expressed as the mean + SD relative to control cells (incubated in the absence of VLDL+IDL). *P < 0.050; **P < 0.010; ***P < 0.001.

Our clinical and *in vivo* findings suggest that lipid oversupply to the myocardium may underlie the observed alterations in serum miR-1 and miR-133a levels. Therefore, we used an *in vitro* model to investigate whether the accumulation of neutral lipids in HL-1 cardiomyocytes induces the release of miR-1 and miR-133a. As previously demonstrated by our group^28, 29^, exposure to VLDL+IDL (50 or 100 μg/mL) dose-dependently increased intracellular neutral lipid content (Fig. 3A). Intracellular neutral lipid accumulation did not induce cardiomyocyte apoptosis (Supplementary Information, Figure S1), in agreement with previous findings of our group^28, 29^.

To determine whether intracellular neutral lipid accumulation influences miRNA release from cardiomyocytes, miRNA levels were analysed in exosomes collected from conditioned medium before and after cardiomyocyte exposure to lipoproteins. The isolated exosomes were subjected to transmission electron microscopy, which revealed small, rounded vesicles (<0.1 μm) surrounded by a bilayered membrane (Supplementary Information, Figure S2A). The exosome marker CD63 was detected by immunoblotting (Supplementary Information, Figure S2B) and the presence of small non-coding RNAs was corroborated (Supplementary Information, Figure S2C). In agreement with previous findings, VLDL+IDL dose-dependently induced the release of miR-1 (27.5-fold and 33.9-fold increases for 50 and 100 μg/mL, respectively) and miR-133a (11.4-fold and 12.6-fold increases for 50 and 100 μg/mL, respectively) from HL-1 cells into the culture medium (Fig. 3B). To further characterize the response of cardiomyocyte-enriched miRNAs to lipid loading, we also evaluated the intracellular profiles of both miRNAs. We observed increased expression of miR-1 (2.4-fold and 2.6-fold for 50 and 100 μg/mL, respectively) and miR-133a (1.3-fold and 1.6-fold for 50 and 100 μg/mL, respectively) after exposure to lipoproteins as compared with control conditions. A dose-dependent response was specifically observed in miR-133a intracellular levels (Fig. 3C).

Given that miRNAs may be packaged in lipoproteins^30, 31^, we analysed the presence of both miR-1 and miR-133a in VLDL+IDL preparations to control for possible cross-contamination in our samples. Neither miR-1, nor miR-133a were detected in the VLDL+IDL preparations added to HL-1 cardiomyocytes.

### Functional analysis

Pathway analysis was performed to identify the biological mechanisms and functions most closely linked with the observed serum miRNA profile. Ten KEGG pathways were enriched with the predicted targets of miR-1 and -133a (p < 0.050) (Supplementary Information, Table S3). A number of pathways related to cardiac function were identified, including the arrhytmogenic right ventricular cardiomyopathy and adrenergic signaling in cardiomyocyte pathways.

## Discussion

Here, we evaluated the expression profile of circulating cardiomyocyte-enriched miRNAs in a population of well-characterized patients with well-controlled type 2 diabetes of short duration and with verified absence of cardiac structural heart disease or inducible ischemia^27^. Quantitative PCR assessment revealed that expression levels of serum miR-1 and miR-133a were directly associated with myocardial steatosis, independently of potential confounding factors. The fact that miR-1 and miR-133a were poorly associated with other clinical, biochemical, metabolic, hemodynamic, and cardiac parameters in regression models supports the hypothesis that these miRNAs are independent predictors of myocardial steatosis. Furthermore, miR-133a levels were higher in patients with uncomplicated type 2 diabetes than in matched healthy subjects, thus paralleling the alterations observed in myocardial steatosis content. Of note, both miRNAs improved the accuracy of a myocardial steatosis model based on clinical parameters including age, plasma fasting glucose, visceral fat volume, non-HDL-cholesterol and plasma triglycerides. The results of patient serum analyses were validated in a murine model of high-fat diet-induced insulin resistance and in an *in vitro* model of lipid-loaded cardiomyocytes. The close association between myocardial steatosis and circulating miR-1 and miR-133a in our population of patients with well-controlled type 2 diabetes levels may be explained, at least in part, by the overaccumulation of neutral lipids in cardiomyocytes. Taken together, our findings suggest that miR-1 and miR-133a are robust predictors of myocardial steatosis in type 2 diabetes.

This study addresses several crucial points relating to circulating miRNA investigations. First, our results show that miRNAs transcribed at the same clusters, such as miR-1 and miR-133a, could be regulated in parallel, at least in response to neutral lipid overaccumulation. Second, increased levels of circulating miR-1 and miR-133a in patients with cardiovascular disease have been previously linked to myocardial ischemia and/or damage^32, 33^. Here, we found a direct association between myocardial steatosis and serum miR-1 and miR-133a levels in type 2 diabetes patients and in a murine model of insulin resistance, even with verified absence of clinically evident myocardial ischemia and/or damage. miRNAs were also detected in exosomes released from HL-1 cardiomyocytes in the absence of cell death. These data are in line with previous reports describing the release of cardiomyocyte-enriched miRNAs in the absence of myocardial damage or necrosis^19^. Our results indicated that miR-1 and miR-133a levels in the systemic circulation may reflect not only myocardial ischemia/damage as previously proposed by other authors^32^, but also active responses of cardiomyocytes to stressful conditions. We and others have demonstrated that hypoxia induces the uptake and accumulation of neutral lipids in cardiomyocytes^28, 34^. Thus, the association observed between the circulating levels of both miRNAs and ischemia could be related, at least in part, to the surrogate ischemia-induced neutral lipid accumulation in the myocardium. Further studies are necessary for exploring this hypothesis. Third, previous studies have described a poor association between circulating miRNAs and clinical outcome after adjustment for routine clinical parameters^35^. Interestingly, the association between serum miRNA levels and myocardial steatosis described here remained statistically significant after adjustment for other potential biomarkers, including well-established cardiac biomarkers such as NT-proBNP and us-CRP^36, 37^. Finally, in agreement with previous reports^38, 39^, serum miRNA levels were independent of most of the clinical characteristics of our population; including typical confounders in type 2 diabetes. Furthermore, except for visceral fat volume, the association between both miRNAs and myocardial steatosis was higher than that observed for other clinical variables. Serum miRNA levels may provide additional information that established clinical parameters cannot. These findings strengthen the clinical applicability of circulating miR-1 and miR-133a as biomarkers of myocardial steatosis in type 2 diabetes patients.

Comparison of our findings with those of other authors is hampered by the paucity of studies that have evaluated the circulating miRNA profile in the context of diabetic cardiomyopathy and cardiac complications in type 2 diabetes. Several studies have proposed circulating miRNAs as useful indicators of vascular complications in diabetic patients^20, 23, 25^. Circulating miR-1 and miR-133 levels have been consistently associated with conditions such as acute coronary syndrome^16, 32, 33, 40^, hypertrophic cardiomyopathy^19^, and Takotsubo cardiomyopathy^18^. However, no previous study has investigated the association between peripheral blood miRNAs and diabetic cardiomyopathy. Our data demonstrate for first time the existence of a specific serum miRNA signature linked to early myocardial dysfunction in patients with well-controlled type 2 diabetes patients and absence of any secondary complication, including diabetes-related vascular complications such as proliferative retinopathy or autonomic neuropathy.

From a clinical perspective, identifying type 2 diabetes subjects with increased risk of incident cardiovascular complications is fundamental for primary prevention and patient management. Early detection and timely management could prevent the development of cardiac-related complications. Although myocardial steatosis precedes the onset of myocardial dysfunction and is evident in the absence of heart failure, its evaluation using ^1^H-MRS in large-scale screenings is impractical. There are currently no soluble biomarkers that can be used to assess neutral lipid accumulation in the heart. Our results suggest that circulating miRNAs may be useful biomarkers for the evaluation of myocardial steatosis severity in patients with uncomplicated type 2 diabetes. Future simplification of the miRNA methodology could allow the development of blood test based on serum miR-1 and miR-133a levels as a non-invasive tool to improve the detection, prediction, and monitoring of cardiac-related complications in the early stages of diabetes. The assessment of expression levels of both miRNAs could allow the identification of high-risk patients in large-scale programs, thus improving cost-effectiveness. Furthermore, the fact that our clinical results could be reproduced in animal models indicates that both miRNAs are suitable for the evaluation of the effectiveness of potential therapeutic interventions.

The active release of miR-1 and miR-133 from HL-1 cardiomyocytes in response to lipid overload and in the absence of cell damage allows us to speculate about the role of both miRNAs as extracellular mediators in cell-cell communication. Myocardial neutral lipid stores are inert and harmless, but reflect the cellular concentrations of cardiotoxic intermediates which alter cardiomyocyte structure and function, leading to cellular stress^41^. miRNAs play a critical role the cellular response to physiologic and pathophysiologic stress^42^, and several studies have demonstrated a role for both miR-1 and miR-133a in cardiac function^43–45^. Our pathways analysis supported this hypothesis. Both miR-1 and miR-133a affect several cellular pathways intimately involved in heart physiology and pathophysiology. Interestingly, one of the benefits conferred by miR-133a in stressed hearts is a reduction in diastolic dysfunction^46^, a hallmark of diabetic cardiomyopathy. Cardiac-specific miR-133a overexpression has also been shown to prevent early cardiac fibrosis in diabetic mice^47^. Functionally competent miRNAs contained within cardiomyocyte exosomes may be delivered to neighbouring cells, where they are capable of regulating gene expression^48^. Indeed, miR-133a-containing exosomes secreted by the H9C2 cardiomyoblast cell line are both transferable and functional^32^. It is thus conceivable that, in conditions of myocardial steatosis, cardiomyocytes release miRNAs into the extracellular space as part of a cellular response induced by neutral lipid overaccumulation. This view is supported by previous studies describing similar protective behaviours in the cardiovascular context. BNP, which is secreted as a prohormone during hemodynamic stress, opposes the physiological abnormalities in heart failure^49^. Furthermore, extracellular vesicles carrying miRNAs from cardiac progenitor cells inhibit apoptosis of mature cardiomyocytes^50^. Our results may further our understanding of the regulatory mechanisms that underlie cardiac-related complications in type 2 diabetes.

Alterations in miRNA signatures have been previously reported in cardiac tissue from type 2 diabetes patients and animal models^51^. In this respect, our results are controversial. We observed an increase in miR-1 and miR-133a expression in the *in vitro* model of lipid-loaded cardiomyocytes. However, expression levels of both miRNAs in lipid-loaded myocardium of high-fat diet-fed mice were similar to those of control mice. Both these findings are in contrast to those of other authors. Conditions linked to diabetic cardiomyopathy, such as cardiac hypertrophy, are inversely associated with the intracellular expression of both miRNAs^43^. In the context of diabetes, the expression of miR-1 and miR-133a are reduced in mouse and rat models of streptozotocin-induced type 1 diabetes^47, 52, 53^. Several factors may account for the discrepancies observed between studies, including the genetic background of the mice used and the protocol employed to induce diabetes. Indeed, alterations in miRNA expression associated with diabetic cardiomyopathy are dependent on the type of diabetes^51^. The complex biology of miRNAs may also influence the respective findings. The miRNA profile of a given cell is highly specific to the stressor which it is exposed and suggests that miRNA expression in the human heart is dynamically regulated as a function of the pathophysiological context^46^.

Strengths of our study are the considerable number of subjects, patients and healthy controls, that have been analysed using ^1^H-MRS, the rigorous control of potential confounding factors, including structural disease and ischemia, and replication using different systems (patients, *in vivo* and *in vitro*), species (human, mice) and sources (serum, culture medium). Nonetheless, some limitations should be noted. Only male patients were studied to avoid possible confounding effects of hormonal status or the use of contraceptives on lipid metabolism in female patients. In addition, it should be noted that the exclusion of type 2 diabetes patients with ischemia or other significant comorbidities precludes generalization of the results. Since the circulating levels of miR-1 and miR-133a have been previously associated with necrotic and ischemic conditions a possible lack of specificity should be considered. Furthermore, we could not discard that other types of cardiac stress, beyond neutral lipid accumulation, could induce the secretion of miR-1 and miR-133a from cardiomyocytes. Finally, these data have yet to be validated in an independent population.

The present study demonstrates for the first time that serum levels of cardiomyocyte-enriched miR-1 and miR-133a are predictors of myocardial steatosis in patients with well-controlled and uncomplicated type 2 diabetes of short duration. These findings could ultimately lead to the use of these miRNAs as novel biomarkers for the diagnosis of subclinical cardiac-related complications in type 2 diabetes patients.

## Methods

### Study subjects

We used samples from the PIRAMID (Pioglitazone Influence on triglyceride Accumulation in the Myocardium In Diabetes) study^27^. Seventy-eight men with uncomplicated type 2 diabetes, aged 45 to 65 years, were deemed eligible. The following inclusion criteria were applied: glycohemoglobin level between 6.5% and 8.5% at screening; BMI between 25 and 32 kg/m^2^; blood pressure <150/85 mmHg (with or without the use of antihypertensive drugs). The exclusion criteria were as follows: any clinically significant disorder, particularly any history or complaints of cardiovascular or liver disease or diabetes-related complications; prior use of thiazolidinediones or insulin. Patient serum samples were analysed before randomization to treatments. Serum samples from 12 male control volunteers of the same age range and BMI were obtained from a previous study^10^. Control subjects fulfilled the following criteria: no known acute or chronic disease based on medical history, physical examination, and standard laboratory tests (blood counts, fasting blood glucose, lipids, serum creatinine, liver enzymes, and electrocardiogram). Exclusion criteria included chronic use of any drug, substance abuse, hypertension, and impaired glucose tolerance (as excluded by a 75-g oral glucose tolerance test). The study was performed at two institutes in The Netherlands (Leiden University Medical Center, Leiden, and Vrije Universiteit Medical Center, Amsterdam) and approved by both local ethics committees. Written informed consent was obtained from all participants. This study was performed in full compliance with the Declaration of Helsinki.

All study procedures and methods have been previously described elsewhere^10, 26, 27^. Serum samples were taken in the morning after an overnight fasting period. Samples were centrifuged and subsequently aliquoted and stored at −80 °C prior to analysis.

### Proton magnetic resonance spectroscopy

Cardiac ^1^H-MRS was performed as described previously^54, 55^. Briefly, myocardial ^1^H-MRS spectra were obtained from the interventricular septum carefully to avoid contamination from epicardial fat. Spectroscopic data acquisition was double-triggered with ECG triggering and respiratory navigator echoes to minimize motion artifacts. Water-suppressed spectra were acquired to measure myocardial triglyceride content, and spectra without water suppression were acquired and used as an internal standard^55^. ^1^H-MRS data were fitted by use of Java-based MR user interface software (jMRUI version 2.2), as described previously^54^. Myocardial steatosis measured as myocardial triglyceride content relative to water was calculated as (signal amplitude of triglyceride)/(signal amplitude of water) ×100^54, 55^.

### Animals

Male C57BL/6 male mice were housed in specific pathogen-free facilities on a 12/12-hour light/dark cycle. Animals (10 weeks of age) were randomized into chow diet (N = 6, 2014 Teklad Global 14% Protein Rodent Maintenance Diet, Harlan Teklad) and high-fat diet (N = 6, TD.88137 Western-type diet, Harlan Teklad) groups. Animals were fed for six weeks. Age-matched male littermates were used for all experiments. All experiments were performed in accordance with relevant guidelines and regulations. All experimental protocols were approved by the Institutional Animal Care and Use Committees of the Institut Català Ciències Cardiovasculars.

Blood samples were taken from the tail and centrifuged at 4 °C and 9,000 g for 10 min to obtain serum. Serum was frozen at −80 °C and used for biochemical analysis and miRNA assessment. Mice were euthanized at week six of the dietetic intervention. Samples of heart and skeletal muscle were frozen at −80 °C for further analysis.

Mice were fasted for 5 hours and serum levels of insulin were measured by a Mouse Insulin ELISA (Mercodia) following manufacturer’s instructions. The HOMA index, an estimation of insulin resistance, was calculated as: [fasting serum insulin (ng/ml) × fasting serum glucose (mM)]/22.5^56^. Serum lipids and lipoproteins, including cholesterol, triglycerides (corrected from free glycerol), phospholipids, NEFAS, HDL cholesterol and phospholipids, were determined enzymatically using commercial kits adapted to a COBAS 6000 autoanalyzer (Roche Diagnostics)^57^.

### Glucose tolerance test

Glucose tolerance test was performed at week four of dietetic intervention. Mice were fasted 5 hours and basal blood glucose level from a tail nick was measured through ACCU-CHEK® Aviva glucometer (Roche). Then, mice were intraperitoneally injected with glucose (1.3 mg/g BW) and blood glucose was then measured at 15, 30, 60, 120 and 180 min after glucose injection. The AUC of the response curve was then calculated with the software Prism 4.0^57^.

### Tissue homogenization

Frozen tissues from mice (25 mg) were pulverized using a mortar and a pestle in liquid nitrogen. Samples were then homogenized in TriPure isolation reagent (Roche) for total RNA according to manufacturer’s instructions.

### Echocardiography in animal model

Transthoracic echocardiography was conducted on all mice under light sedation (1% isoflurane in oxygen) after 6 weeks of western-type or chow diets. Echocardiography was performed using an 18 to 38 MHz linear-array transducer with a digital ultrasound system (Vevo 2100 Imaging System, Visual Sonics). Images were obtained in B-mode and M-mode in the parasternal short-axis views. All images were acquired and measured by an investigator who was blinded to the treatment groups. Standard parameters were measured including left ventricle (LV) end-diastolic diameter (LVDd) and LV end-systolic diameter (LVDs), LV end-diastolic volume (LVEDV), LV end-systolic volume (LVESV) and LV ejection fraction (EF).

### HL-1 cardiomyocyte cell culture

The murine HL-1 cell line was generated by Dr. W.C. Claycomb (Louisiana State University Medical Centre, New Orleans, Louisiana, USA) and kindly provided by Dr. U Rauch (Charité-Universitätsmedizin Berlin). These cells show cardiac characteristics similar to those of adult cardiomyocytes^58^. HL-1 cells were maintained in Claycomb medium (JRH Biosciences, Lenexa) supplemented with 10% fetal bovine serum (FBS) (Invitrogen Corporation), 100 mM norepinephrine, 100 units/mL penicillin, 100 mg/mL streptomycin, and 2 mM L-glutamine (Sigma Chemical Company) in plastic dishes coated with 12.5 mg/mL fibronectin and 0.02% gelatine, and in a 5% CO_2_ atmosphere at 37 °C. Cells were arrested during 24 h and incubated in absence or presence of lipoproteins as previously described^28^.

### Lipoprotein isolation

Human very-low density and intermediate density lipoproteins VLDL+IDL (d_1.001_ − d_1.019_ g/mL) were obtained from pooled sera of healthy normolipemic donors who gave their written informed consent by sequential ultracentrifugation^59^. Lipoprotein preparations used in the experiments were less than 24 hours old and have no detectable levels of endotoxin (Limulus AmebocyteLysate test, Bio Whittaker).

### Isolation of Exosomes

Exosomes were purified from conditioned medium of Hl-1 cardiomyocytes by several centrifugation steps. Briefly, supernatant was centrifuged at 400 × g for 15 min at 4 °C, 1,200 × g for 20 min at 4 °C, 12,500 × g for 5 min at 4 °C to eliminate floating cells, cellular nuclei, cell debris and apoptotic bodies. For exosome purification the supernatant was centrifuged at 120,000 × g for 120 min at 4 °C, followed by an additional washing step at 120,000 × g for 120 min at 4 °C. The pelleted vesicles were resuspended in PBS. The isolated exosomes were subjected to transmission electron microscopy and immunoblotting of exosome markers (CD63). Detection of RNA was performed using Agilent 2100 Bioanalyzer® (Agilent).

### Determination of neutral lipid content

HL-1 cardiomyocytes were exhaustively washed and harvested in NaOH 0.1 M following the VLDL+IDL incubation period. In the animal experimental model, one portion of myocardial tissue (5 mg) was also homogenized in NaOH 0.1M. Neutral lipids from HL-1 cardiomyocytes and mouse myocardium were extracted as previously described^28^ and cholesteryl esters, free cholesterol and triglyceride content were analysed by thin layer chromatography. The organic solvent was removed under N_2_ stream, the lipid extract was redissolved in dichloromethomethane and one aliquot was partitioned by thin layer chromatography (TLC). TLC was performed on silica G-24 plates. The different concentrations of standards (a mixture of cholesterol, cholesterol palmitate and triglycerides) were applied to each plate. The chromatographic developing solution was heptane/diethylether/acetic acid (74:21:4, v/v/v). The spots corresponding to cholesteryl esters, free cholesterol and triglycerides were quantified by densitometry against the standard curve of cholesterol palmitate, cholesterol and triglycerides, respectively, using a computing densitometer (Molecular Dynamics).

### Quantification of microRNAs

According to manufacturer’s instructions different kits were used for extracellular or intracellular miRNA isolation. Total RNA extraction was performed using the mirVana PARIS kit (Thermo Fisher) for serum and exosomes and the mirVana miRNA Isolation kit (Thermo Fisher) for HL-1 cardiomyocytes. RNA spike-in kit (Exiqon) was used in all extractions to determine the quality of the sample. For serum and exosomes samples, synthetic *Caenorhabditis elegans* miR-39-3p (cel-miR-39-3p) was spiked into samples as an external standard after incubation with the denaturing solution. The mixture was also supplemented with 1 μg of MS2 carrier RNA (Roche) to improve miRNA yield, as recommended by the manufacturer. cDNA was synthesized using the universal cDNA synthesis kit II (Exiqon). Different protocols of cDNA synthesis were used for intracellular or extracellular miRNAs, according to the manufacturer’s instructions. For HL-1 cardiomyocytes, total RNA concentration was determined with a NanoDrop ND-1000 spectrophotometer (NanoDrop Technologies). Then, RNA samples were adjusted to a concentration of 5 ng/μL using nuclease free water. The amount of RNA present in serum and exosomes could not be accurately determined. Thus, we used the same starting sample volume rather than RNA quantity. All starting volumes were homogeneous according the type of sample. miRNAs were quantified by quantitative real time polymerase chain reaction (qPCR) using the ExiLENT SYBR Green master mix (Exiqon) and a 7900HT Fast Real-Time PCR System (Applied Biosystems). Each experiment was performed with two technical replicates. A CV < 20% cutoff was established for validating the technical replicates. Quantitative miRNA analysis was restricted to cardiomyocyte-enriched miRNAs: miR-1, miR-133a/b, miR-208a/b, and miR-499. Small nuclear U6 and small nucleolar RNA SNORD48 were examined as internal normalization controls, as recommended by the manufacturer. For HL-1 cardiomyocytes and myocardium, U6 was detectable in all samples and presented low dispersion, and therefore, was used for normalization. For serum and exosome samples, no internal control was suitable for normalization, and thus, cel-miR-39-3p was used for normalization. Relative quantification was performed using the 2^−dCt^ method, where dCt = mean Ct_target_ − mean Ct_U6_ for HL-1 cardiomyocytes and myocardium; and dCt = mean Ct_target_ − mean Ct_cel-miR-39_ for serum and exosome samples. Influence of haemolysis was discarded by analysing the Ct values of miR-23a and miR-451a^60^.

### Pathway analysis

Pathway analysis was performed as previously described by our group^61^, using the web-based computational tool DIANA-miRPath v3.0^62^. DIANA-miRPath v3.0 utilizes predicted miRNA targets from the DIANA-microT-CDS algorithm and combines the results with the pathway tool KEGG (Kyoto Encyclopedia of Genes and Genomes) to identify possible targets. The level of significance was set at *P* < 0.050.

### Statistical analysis

The statistical software package SPSS 15.0 for Windows (SPSS Inc.) was used for all statistical analyses. Descriptive statistics were used to characterize study populations and to analyse the studied parameters. Data were presented as the mean ± SD for continuous variables and as frequencies (percentages) for categorical variables. The normality of the data was analysed using the Kolmogorov-Smirnov test. Variables with skewed distribution were log-transformed prior to use as continuous variables in statistical analyses. Continuous variables were compared between groups using a Student’s t-test for independent samples or a one-way ANOVA followed by Tukey’s *post hoc* test for comparison between each subgroup. Correlations between variables were analysed using Pearson’s correlation analysis. The results are presented using Pearson’s correlation coefficient (ρ). Univariate linear regression was performed to examine the associations between myocardial steatosis or circulating cardiomyocyte-enriched miRNA levels and possible predictors. To identify independent predictors of myocardial steatosis in multivariate analyses, myocardial steatosis was entered into the models as a dependent variable and circulating cardiomyocyte-enriched miRNA, age, visceral fat volume, non-HDL-cholesterol, and plasma TG were subsequently entered as independent variables (model 2). In addition, possible confounding variables such as plasma fasting glucose, plasma fasting insulin, BMI, HDL-cholesterol, plasma NEFA, NT-proBNP, us-CRP, LV mass, ejection fraction, and E/E_a_ were separately entered into the models (model 3). Data are expressed as the standardized beta coefficient (β). Univariate and multivariate logistic regressions were analysed to explore the association between serum levels of miR-1 and miR-133a, and myocardial steatosis as outcome. Patients with type 2 diabetes were dichotomized into two categories according to myocardial tertiles: tertiles 1 and 2 (low-intermediate myocardial steatosis) and tertile 3 (high myocardial steatosis). In order to establish whether the association between myocardial steatosis and circulating miRNAs levels could be influenced by possible confounders, associations were also adjusted for age, plasma fasting glucose, visceral fat volume, non-HDL-cholesterol and plasma triglyceride (model 1). The results are presented as odds ratios (OR) and 95% confidence intervals (CI). AUC was analysed to explore the accuracy of the logistic regression models. Bonferroni-Holm test was performed to correct for multiple comparisons. Differences were considered statistically significant when *P* < 0.050.

## Electronic supplementary material

Supplementary Information
