# Supplementary material for: Serum microRNA-1 and microRNA-133a levels reflect myocardial steatosis in uncomplicated type 2 diabetes
Source: Sci Rep. 2017 Mar 3;7:47. doi: 10.1038/s41598-017-00070-6 (PMC5428350; doi:10.1038/s41598-017-00070-6)
Supplement: Supplementary file 1 — Supplementary Information [file 41598_2017_70_MOESM1_ESM.doc]

**Serum microRNA-1 and microRNA-133a levels reflect myocardial steatosis in uncomplicated type 2 diabetes**

de Gonzalo-Calvo D,^1*^ van der Meer RW,^2^ Rijzewijk LJ,^3^ Smit JWA,^4^ Revuelta-Lopez E,^1^ Nasarre L,^1^ Escola-Gil JC,^5^ Lamb HJ,^2^ Llorente-Cortes V^1,6*^.

^1^ Cardiovascular Research Center, CSIC-ICCC, Biomedical Research Institute Sant Pau (IIB Sant Pau), Barcelona, Spain

^2^ Department of Radiology, Leiden University Medical Center, Leiden, The Netherlands

^3^ Department of Medicine, Kantonsspital Baden AG, Baden, Switzerland

^4^ Department of Internal Medicine, University Medical Center Nijmegen, Nijmegen, The Netherlands

^5^ IIB Sant Pau, Departament de Bioquímica i Biologia Molecular, Universitat Autònoma de Barcelona-CIBER de Diabetes y Enfermedades Metabolicas Asociadas, Barcelona, Spain

^6^ Biomedical Research Institute of Barcelona, CSIC.

**Corresponding authors*:**

**David de Gonzalo Calvo, PhD**

Cardiovascular Research Center, CSIC-ICCC, Biomedical Research Institute Sant Pau (IIB Sant Pau), Av. Sant Antoni Maria Claret 167, Pavelló del Convent, 08025 Barcelona, Spain.
Tel: +34 93 556 5901 Fax: +34 935565559

E-mail: [DGonzalo@santpau.cat](mailto:DGonzalo@santpau.cat)

**Vicenta Llorente Cortés, PhD**

Cardiovascular Research Center, CSIC-ICCC, Biomedical Research Institute Sant Pau (IIB Sant Pau), Av. Sant Antoni Maria Claret 167, Pavelló del Convent, 08025 Barcelona, Spain.
Tel: +34 93 556 5888, Fax: +34 935565559

E-mail: [cllorente@csic-iccc.org](mailto:cllorente@csic-iccc.org)

**SUPPLEMENTARY INFORMATION**

| **Table S1. Characteristics of the study groups.** | |  |  |
| --- | --- | --- | --- |
|  |  |  |  |
| **Variable** | **HEALTHY GROUP** | **TYPE 2 DIABETES GROUP** | ***P*-value** |
|  |  |  |  |
| N | 12 | 12 |  |
| Male N (%) | 12 (100) | 12 (100) |  |
| Age (years) | 57.7 ± 6.7 | 58.5 ± 6.2 | 0.763 |
| Body mass index (kg/m^2^) | 26.8 ± 2.1 | 27.5 ± 2.2 | 0.480 |
| Plasma fasting glucose (mmol/L) | 5.3 ± 0.4 | 9.6 ± 2.5 | <0.001* |
|  |  |  |  |
| Myocardial steatosis (%) | 0.7 ± 0.2 | 1.1 ± 0.6 | 0.027* |
|  |  |  |  |
| Data are presented as mean ± SD. | |  |  |
| For abbreviations see the text. | |  |  |

| **Table S2. Characteristics of the western diet-fed and chow diet-fed groups.** | | | |
| --- | --- | --- | --- |
|  |  |  |  |
| **Variable** | **Chow** | **Western** | ***P*-value** |
|  |  |  |  |
| N | 6 | 6 |  |
| Weight (g) | 34.3 ± 1.1 | 36.2 ± 1.5 | 0.029* |
| Serum triglycerides (mM) | 1.0 ± 0.2 | 0.6 ± 0.2 | 0.746 |
| Serum total cholesterol (mM) | 3.7 ± 0.6 | 6.2 ± 0.3 | <0.001* |
| Serum HDL-cholesterol (mM) | 3.5 ± 0.4 | 5.2 ± 0.4 | <0.001* |
| Serum NEFAs (mM) | 1.1 ± 0.4 | 0.9 ± 0.1 | 0.521 |
|  |  |  |  |
| Data are presented as mean ± SD. | | | |

| **Table S3. KEGG pathways.** | | |
| --- | --- | --- |
|  |  |  |
| **KEGG Pathway** | **P-value** | **No. Of Genes** |
|  | | |
| Glycosphingolipid biosynthesis - lacto and neolacto series (hsa00601) | 3.07x10^-6^ | 2 |
| ECM-receptor interaction (hsa04512) | 7.12 x10^-6^ | 9 |
| Gap junction (hsa04540) | 0.00282 | 12 |
| Transcriptional misregulation in cancer(hsa05202) | 0.00282 | 26 |
| Bacterial invasion of epithelial cells (hsa05100) | 0.00925 | 13 |
| Amphetamine addiction (hsa05031) | 0.00953 | 11 |
| Morphine addiction (hsa05032) | 0.00953 | 12 |
| Arrhytmogenic right ventricular cardiomyopathy (ARVC) (hsa05412) | 0.00953 | 7 |
| Adrenergic signaling in cardiomyocytes (hsa04261) | 0.01052 | 15 |
| Lysine degradation (hsa00310) | 0.01948 | 7 |
|  |  |  |

**Supplementary Figures**

**
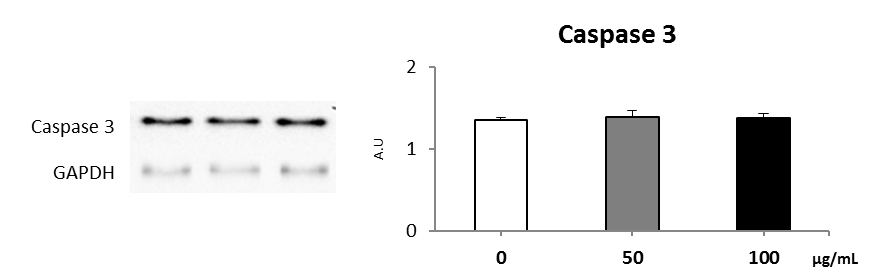
**

**Supplementary Figure S1**. Caspase 3 levels in HL-1 cardiomyocytes after exposition to VLDL+IDL. HL-1 cardiomyocytes were incubated in the absence or presence of VLDL+IDL (50 and 100 µg/mL) for 24 hours. Representative Western blot showing caspase 3 and GAPDH bands. Bar graphs showing the mean ± SD of caspase 3 levels. Cropped blots are displayed.

**
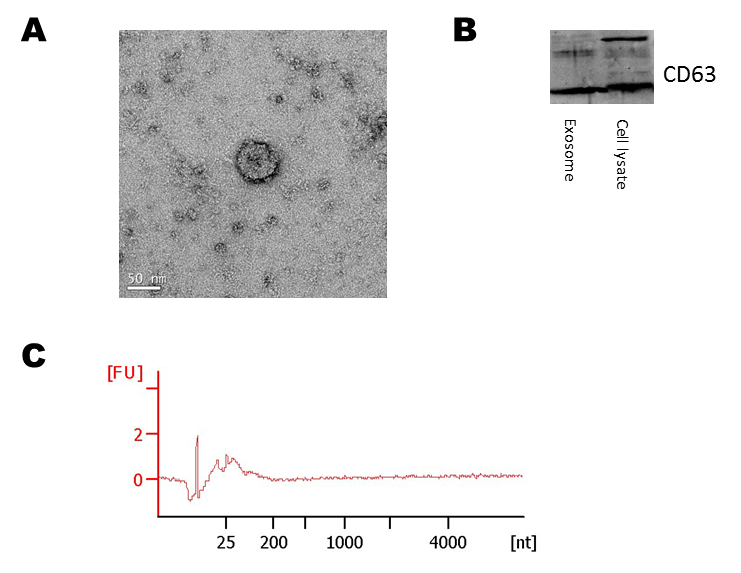
**

**Supplementary Figure S2.** A) Transmission electronic microscopy of purified HL-1 exosomes. Scale bar = 50 nm; B) Western blot, Anti-CD63, Purified HL-1 exosomes and HL-1 cell lysates. Cropped blots are displayed; C) Bioanalyzer Pico Analysis of purified exosomes total RNA

| 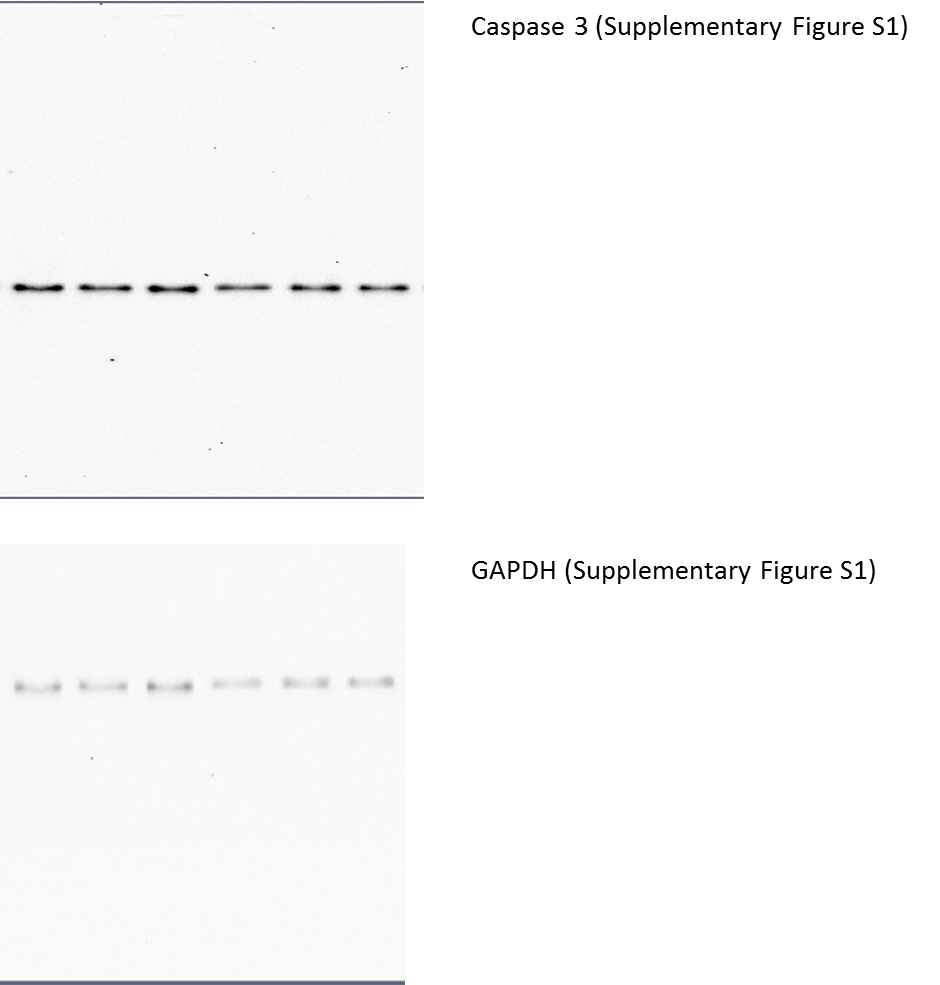 |
| --- |
| **Supplementary Figure S3.** Full-length gels. |

.
